# Association of the Modified Mediterranean Diet Score (mMDS) with Anthropometric and Biochemical Indices in US Career Firefighters

**DOI:** 10.3390/nu12123693

**Published:** 2020-11-30

**Authors:** Maria Romanidou, Grigorios Tripsianis, Maria Soledad Hershey, Mercedes Sotos-Prieto, Costas Christophi, Steven Moffatt, Theodoros C. Constantinidis, Stefanos N. Kales

**Affiliations:** 1 Department of Medical Statistics, Medical Faculty, Democritus University of Thrace, 68100 Alexandroupolis, Greece; gtryps@med.duth.gr; 2 Department of Preventive Medicine and Public Health, Navarra Institute for Health Research, University of Navarra, 31008 Pamplona, Spain; mhershey@alumni.unav.es; 3 Department of Environmental Health, T.H. Chan School of Public Health, Harvard University, Boston, MA 02215, USA or Mercedes.sotos@uam.es (M.S.-P.); costas.christophi@cut.ac.cy (C.C.); skales@hsph.harvard.edu (S.N.K.); 4 Department of Preventive Medicine and Public Health, School of Medicine, Universidad Autónoma de Madrid, IdiPaz (Instituto de Investigación Sanitaria Hospital Universitario La Paz), Calle del Arzobispo Morcillo 4, 28029 Madrid, Spain; 5 Biomedical Research Network Centre of Epidemiology and Public Health (CIBERESP), Carlos III Health Institute, 28029 Madrid, Spain; 6 Cyprus International Institute for Environmental and Public Health, Cyprus University of Technology, 30 Archbishop Kyprianou Str., Lemesos 3036, Cyprus; 7 National Institute for Public Safety Health, IN 324 E New York Street, Indianapolis, IN 46204, USA; steven.moffatt@ascension.org; 8 Laboratory of Hygiene and Environmental Protection, Medical School, Democritus University of Thrace, 68100 Alexandroupolis, Greece; tconstan@med.duth.gr; 9 Occupational Medicine, Cambridge Health Alliance/Harvard Medical School, Cambridge, MA 02319, USA

**Keywords:** Mediterranean diet, Mediterranean diet scores, anthropometrics, lipids, cardiometabolic risk

## Abstract

The Mediterranean diet is associated with multiple health benefits, and the modified Mediterranean Diet Score (mMDS) has been previously validated as a measure of Mediterranean diet adherence. The aim of this study was to examine associations between the mMDS and anthropometric indices, blood pressure, and biochemical parameters in a sample of career firefighters. The participants were from Indiana Fire Departments, taking part in the “Feeding America’s Bravest” study, a cluster-randomized controlled trial that aimed to assess the efficacy of a Mediterranean diet intervention. We measured Mediterranean diet adherence using the mMDS. Anthropometric, blood pressure, and biochemical measurements were also collected. Univariate and multivariate linear regression models were used. In unadjusted analyses, many expected favorable associations between the mMDS and cardiovascular disease risk factors were found among the 460 firefighters. After adjustment for age, gender, ethnicity, physical activity, and smoking, a unitary increase in the mMDS remained associated with a decrease of the total cholesterol/HDL ratio (β-coefficient −0.028, *p* = 0.002) and an increase of HDL-cholesterol (β-coefficient 0.254, *p* = 0.004). In conclusion, greater adherence to the Mediterranean diet was associated with markers of decreased cardiometabolic risk. The mMDS score is a valid instrument for measuring adherence to the Mediterranean diet and may have additional utility in research and clinical practice.

## 1. Introduction

Obesity, metabolic syndrome, and cardiovascular disease (CVD) have major impacts on US emergency responders, such as firefighters. These non-communicable, lifestyle-influenced conditions can put firefighters’ career and life at risk [1,2,3,4,5,6]. The hazardous working environment, with risks of burns and physical trauma, air pollutants, physical and emotional stress, and shiftwork, causes additional stress to the cardiovascular system and may put firefighters at a higher cardiovascular disease risk with respect to the general population [7,8]. In fact, among US firefighters, sudden cardiac death is the leading cause of on-duty death, is, in most cases, due to underlying coronary heart disease and cardiomegaly, and is responsible for over 40% of duty-related deaths [9,10].

The number and proportion of CVD fatalities have remained relatively similar over the years, which suggests the need for more aggressive lifestyle-related interventions [11]. A recent study in older firefighters suggested that wellness programs can improve the cardiorespiratory function [12]. The eating and lifestyle patterns of firefighters often lead to obesity and have negative impacts on society by contributing to an increased rate of sick leave and increased healthcare expenses [13,14]. On the other hand, firefighters who follow a healthy lifestyle by exercising and maintaining a healthy weight are more likely to maintain high levels of cardiorespiratory fitness during aging [15].

The Mediterranean diet has been shown to reduce the risk of CVD and promote longevity in a variety of international settings [16,17,18]. There is also an increasing trend to introduce the Mediterranean diet at work as an intervention to prevent non-communicable diseases [19]. The existing evidence also suggests that adherence to the Mediterranean diet not only improves the physical health and wellbeing of workers but also may reduce work stress and blood pressure [20,21,22]. In two recent meta-analyses of Randomized Control Trials (RCT), the Mediterranean diet, also in combination with physical activity, was the only eating pattern which showed significant and beneficial effects on weight, body mass index (BMI) waist circumference, total cholesterol, high-density lipoprotein (HDL)-cholesterol, glucose, and blood pressure, without any evidence of adverse associations [23,24].

Various Mediterranean diet scores have been developed worldwide to quantify adherence to the Mediterranean diet [25,26,27,28,29]. In the ATTICA intervention in Greece, adherence was measured with the MedDietScore (0–55 items) [30,31], while the European Prospective Investigation into Cancer Nutrition (EPIC) group used the MED score (0–9 scale) [32,33]. Other adaptations include the Italian alternative Mediterranean diet score aMED (0–9 scale) [34] and the I-MEDAS from Israel (17-item questionnaire) [35]. The PREDIMED score, which is based on 14 items from the Prevención con Dieta Mediterránea in Spain, is also widely used [36,37]. However, the use of these scores in different populations, cultures, and ethnicities has been questioned, as they may not be directly adaptable to different ethnic and social groups [38].

In the US, a Mediterranean diet score was constructed specifically to measure adherence to the Mediterranean diet in career firefighters and is known as the modified Mediterranean Diet Score (mMDS) [39].

“Feeding America’s Bravest” is a cluster-randomized-controlled trial that aimed to assess the efficacy of a Mediterranean Diet intervention in 60 fire stations in two Indiana (USA) Fire Departments [40]. To assess Mediterranean diet adherence, the aforementioned mMDS was used. Its validity versus previously validated questionnaires [41,42] was established using a sample of firefighters participating in “Feeding America’s Bravest” [43]. The aim of the present study was to further corroborate the validity of the mMDS as a measure of Mediterranean diet adherence by examining its cross-sectional associations with anthropometric indices, blood pressure, and biochemical parameters in participants of the “Feeding America’s Bravest” study.

## 2. Materials and Methods 

### 2.1. Study Population 

**Figure 1:**
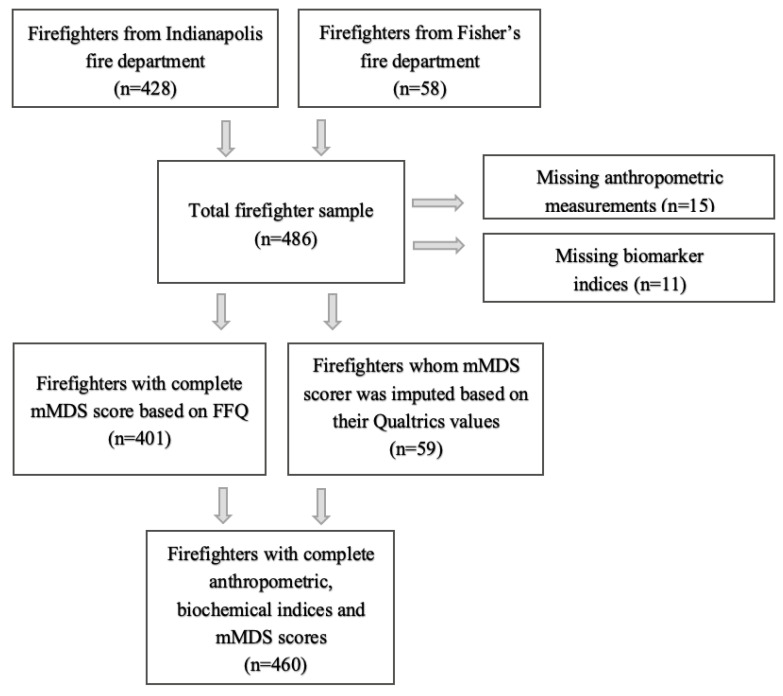
Flow chart for firefighters’ sample selection. mMDS: modified Mediterranean Diet Score, FFQ: Food Frequency Questionnaire.

In this cross-sectional study, we used baseline nutrition surveys to calculate the mMDS from a total study base of 486 career firefighters (428 firefighters were recruited from the Indianapolis Fire Department’s 44 stations and 58 from the Fishers, Indiana Fire Department’s 6 stations) who consented to and enrolled in the ongoing study “Feeding America’s Bravest”: Mediterranean Diet-Based Interventions to change Firefighters’ Eating Habits and Improve Cardiovascular Risk Profiles between 28 November 2016 and 16 April 2018 [34,39]. We excluded firefighters who did not complete baseline anthropometric measurements or if their biomarker indices were missing (Figure 1). Recruitment, consent, and study procedures were carried out by trained staff of the National Institute for Public Safety Health, who work regularly with both or the respective fire departments.

### 2.2. Dietary Assessment

A validated 131-item semi-quantitative Food Frequency Questionnaire (FFQ) [41] and the mMDS score [39] were used to quantify the firefighters’ dietary intake patterns at baseline. The FFQ is a questionnaire previously developed by Yang et al. [39]. Two additional domains were added to the mMDS (nuts and legume consumption), and the score ranged between 0 = minimum adherence to the Mediterranean diet and 51 = maximum adherence to the Mediterranean diet. Because we had previously validated the mMDS in a Qualtrics survey with the Harvard FFQ [43] and more initial Indianapolis participants had complete FFQs, we calculated their mMDS score based on the FFQ and used a scaled value of the directly derived Qualtrics score for the Fishers firefighters who had not done an FFQ.

### 2.3. Physical Activity

Physical activity was calculated based on a 0–7 scale through a validated self-report scale (Self-Report of Physical Activity (SRPA)) embedded into our study questionnaire [44]. At baseline, the firefighters were asked to describe their physical activity levels over the past month using the following options: 0 = avoid walking or exertion (e.g., always use elevator, drive whenever possible instead of walking, biking, or rollerblading); 1 = walk for pleasure, routinely use stairs, occasionally exercise sufficiently to cause heavy breathing or perspiration; 2 = 10 to 60 min per week; 3 = over one hour per week; 4 = run less than 1 mile per week or spend less than 30 min per week in comparable physical activity; 5 = run 1 to 5 miles per week or spend 30 to 60 min per week in comparable physical activity; 6 = run 5 to 10 miles per week or spend 1 to 3 h per week in comparable physical activity; and 7 = run over 10 miles per week or spend over 3 h per week in comparable physical activity.

### 2.4. Outcome Assessments

At baseline recruitment, the participants underwent blood pressure and anthropometric assessments as part of the initial study visit. Resting blood pressure was measured using an appropriately sized cuff in seated position for each firefighter. BMI was recorded for all study subjects in kg/m^2^ from measured height and weight. Body fat (%) was estimated by a Bioelectrical Impedance Analyzer (BIA) [40,45].

Separately, the firefighters had biochemical indices assessed at fire department-sponsored medical examinations. We used the biochemical measurements gathered at the closest date from the date of study consent within the same 12-month period. Blood samples were also collected after an overnight fast at baseline and at follow-up. Using EDTA collection tubes, up to 15 mL of blood was collected. Plasma and serum were aliquoted, frozen at −80 °C, stored, and run in batches. Automated high-throughput enzymatic analysis was used to determine the blood lipid profiles of the firefighters. This analysis achieved coefficients of variation ≤3% for cholesterol and ≤5% for triglycerides, using a cholesterol assay kit and reagents (Ref:7D62–21) and triglyceride assay kit and reagents (Ref:7D74–21) by the ARCHITECT c System, Abbott Laboratories, IL, USA. The lipid measures included total cholesterol, triglycerides, total cholesterol/HDL ratio, HDL-cholesterol, and low-density lipoprotein (LDL)-cholesterol.

### 2.5. Covariate Assessment

We collected sociodemographic characteristics, medical history, lifestyle habits, and dietary intake from the study’s comprehensive lifestyle questionnaire [40].

### 2.6. Statistical Analysis

Statistical analysis was performed using IBM Statistical Package for the Social Sciences (SPSS), version 19.0 (IBM Corp., Armonk, NY, USA). The normality of the quantitative variables was tested with the Kolmogorov-Smirnov test. The quantitative variables were expressed as mean ± standard deviation (SD) or as median (Q1, Q3), as appropriate. The qualitative variables were expressed as absolute and relative (%) frequencies.

Multivariable linear regression models were used to examine the association of mMDS with anthropometric, blood pressure, and biochemical variables, after adjusting for age, gender, race, physical activity, and smoking. Beta coefficients were reported with the corresponding standard errors (SE) and *p* values. Component items of the mMDS were compared between firefighters with high and low values of biochemical parameters using the chi-square test.

All tests were two-tailed, and statistical significance was considered for *p* values < 0.05.

### 2.7. Ethics Statement

The overarching “Feeding America’s Bravest” protocol was approved by the Harvard Institutional Review Board (IRB16-0170) ethics committee and is registered at Clinical Trials (NCT02941757). All participants provided signed informed consent for participation. The participants who met the criteria for enrollment in the intervention were all informed about their right to decline participation to the intervention or to withdraw at any time as per the Declaration of Helsinki, and the participants who decided to enroll gave full informed consent as per the protocol of the research [40].

## 3. Results

### 3.1. Sampling Procedure and Outcome

A sample of 460 firefighters from the two fire departments had complete data for analysis in the current study and represented 95% of all participants who consented to the parent clinical trial (Figure 1).

### 3.2. General Characteristics of the Firefighters

The majority of the firefighters were males (94.4%), with a mean age of 46.7 years (SD 8.3 years). Firefighters’ personal characteristics are shown in Table 1. The mean mMDS in the study population was 21.88 (SD 6.68). The majority of the firefighters were overweight/obese, with an average body fat percentage of 28.10% (SD 6.55%).

### 3.3. Association of the Modified Mediterranean Diet Score with Anthropometric and Biochemical Indices

The association of mMDS with the participants’ anthropometric measures, blood pressure, and biochemical variables is shown in Table 2. When the mMDS scores were categorized into quartiles, multivariate analysis adjusted for age and gender revealed statistically significant inverse associations of mMDS quartiles with BMI (*p* = 0.030), waist circumference (*p* = 0.002), body fat percentage (*p* = 0.002), and total cholesterol/HDL ratio (*p* = 0.007), whereas there was a positive association with HDL-cholesterol (*p* = 0.002).

After further adjustment for subjects’ ethnicity, physical activity, and smoking (Table 2), being in a higher mMDS quartile remained significantly inversely associated with the total cholesterol/HDL ratio (*p* = 0.020) and positively associated with HDL-cholesterol (*p* = 0.022).

### 3.4. Effects of a Unitary Increase in the Modified Mediteranean Score on Anthropometric Measures, Blood Pressure, and Biochemical Indices

The association of mMDS with subjects’ anthropometric measures, blood pressure, and biochemical variables, as a continuous variable, was further analyzed using linear regression models (Table 3).

Multivariate linear regression analysis, adjusting for subjects’ age and gender, revealed that a unitary increase in the mMDS was significantly inversely associated with BMI (β-coefficient −0.080, *p* = 0.008), waist circumference (β-coefficient −0.114, *p* < 0.001), body fat percentage (β-coefficient −0.141, *p* = 0.001), and total cholesterol/HDL ratio (β-coefficient −0.028, *p* = 0.002), whereas it was positively associated with HDL-cholesterol (β-coefficient 0.254, *p* < 0.001). After further adjustment for subjects’ ethnicity, physical activity, and smoking, mMDS was significantly associated with a lower total cholesterol/HDL ratio (β-coefficient −0.030, *p* = 0.002), whereas there was a positive association of mMDS with HDL-cholesterol (β-coefficient 0.286, *p* = 0.004).

### 3.5. Effects of Single Components of the Modified Mediteranean Score on Anthropometric Measures, Blood Pressure, and Biochemical Indices

Examining component food items of the mMDS and total cholesterol/HDL ratio, total cholesterol-HDL ratio, and blood glucose, fast-food consumption was positively associated with a total cholesterol/HDL ratio >6 (*p* = 0.003) and with triglycerides levels ≥150 mg/dL (*p* < 0.001). Sweet desserts consumption was associated with a total cholesterol/HDL ratio >6 (*p* = 0.004) and with triglycerides levels ≥150 mg/dL (*p* = 0.002), while lower consumption of fruits and vegetables was associated with a total cholesterol/HDL ratio >6 (*p* = 0.049). Fried food consumption was associated with a total cholesterol/HDL ratio >6 (*p* = 0.004) and with triglycerides levels ≥150 mg/dL (*p* = 0.037), and consumption of non-alcoholic beverages at home was associated with glucose levels ≥100 mg/dL (*p* = 0.036). No other statistically significant associations were observed (Appendix A Table A1).

## 4. Discussion

Our study shows that greater adherence to a Mediterranean diet, as measured by higher mMDS, was favorably associated, as expected, with various anthropometric and biochemical parameters after adjustment by age and gender. After further adjustment for ethnicity, physical activity, and smoking, a higher mMDS remained associated with a lower total cholesterol/HDL ratio and increased HDL-cholesterol. These results are generally in agreement with those of our previous larger study in a different Midwest firefighter cohort of 780 career male firefighters. The study sample was representative, as the participants had similar demographics, anthropometrics, and dietary habits to those of their entire fire departments and other mid-Western firefighters [39]. In our former cross-sectional study, the results indicated that a higher mMDS was associated with HDL-cholesterol and with lower LDL-cholesterol when adjusted for age, BMI, and physical activity and that the firefighters who adhere the most to the Mediterranean diet had a 35% lower risk of prevalent metabolic syndrome [39]. Taken together, our findings are biologically plausible based on previous research and lend additional credibility and validity to the mMDS. The PREDIMED study also found similar results for the Mediterranean diet arms of the intervention, where a reduction of carbohydrates and the increase of monounsaturated dietary fatty acids (MUFA) resulted in lower cholesterol levels and increased HDL cholesterol levels [46]. Similar results were reported from another recent randomized control trial from Italy [47]. In summary, the present study is consistent with past research demonstrating that the Mediterranean diet has cardioprotective effects by improving HDL-cholesterol levels and the total cholesterol/HDL ratio [19,23,48].

Regarding anthropometrics, our results adjusted for age and gender were consistent with previous findings associating the Mediterranean diet with BMI, waist circumference, and weight loss [19,34,49,50,51,52]. However, we found no statistically significant associations, after further adjusting for ethnicity, physical activity, and smoking status. Similarly, several other scores such as the Mediterranean Diet Scale (MDScale), Mediterranean Food Pattern (MFP), MD Score (MDS), Short Mediterranean Diet Questionnaire (SMDQ), and MedDiet score were also not significantly associated with BMI [51,52]. The difference between our unadjusted and adjusted models may indicate an insufficient sample size in the current study.

In our study, there was no statistically significant association between the mMDS and glucose levels, consistent with previous research and the most recent RCT meta-analysis studies [23,24,53], although we did find that high consumption of non-alcoholic sugar-sweetened beverages at home was associated with higher glucose levels, as has been shown elsewhere [54,55]. Sweet desserts consumption was associated with a total cholesterol/HDL ratio >6 and with triglycerides levels ≥150 mg/dL. Firefighters with low fruit consumption were more likely to have a total cholesterol/HDL ratio >6. On average, the firefighters were consuming three servings of fruits and vegetables per day, in contrast with the recommendations of five or more daily servings of fruits and vegetables of the American Heart Association (AHA) [56]. Thus, our results highlight the need to increase the consumption of fruits and vegetables, because of their cardioprotective role, as an integral part of the Mediterranean diet [48,57,58]. In a recent study based on how the American population can adopt the Mediterranean diet, it was recommended that the American population should replace their usual desserts such us cookies, ice creams, pies, and sweet and creamy desserts with fresh fruits to optimize their health [59]. Increased fried food consumption was also associated with a total cholesterol/HDL ratio >6 and with triglycerides levels ≥150 mg/dL. It is well documented that the quality of fried food depends on the type of the oil used for frying [60]. Even though the scores for cooking with oils or fats at home and at work were not associated with any of the indices, these scores were below 4, indicating that the consumed fat or oils were mostly oils and spreads other than olive oil (e.g., margarine, corn or vegetable oil, and other spreads). Because at baseline the firefighters were unlikely to use olive oil for cooking, their olive oil consumption was reduced, and they were missing a basic component of the Mediterranean diet which is very important for its anti-inflammatory and antioxidant benefits [61,62,63].

The major limitation of this study is its cross-sectional nature, which does not allow us to infer causation. Another limitation of our study is that the firefighters were mainly men (94.4%). However, this reflects the current demographic of the US career fire service. Our study was also subject to a degree of non-response bias, as the lifestyle questionnaires were completed online by firefighters and not during the face-to-face study visits.

One of our study’s strengths is that the firefighters’ anthropometrics included their body fat percentage and waist circumference, not only their BMI. In fact, BMI may cause some false positives due to the increased muscle mass of some firefighters [64]. Another strength is that all our data were collected using standardized procedures, which limits bias. Also, the mMDS was created so to cover the eating habits of the firefighters at work and at home for better accuracy [39]. Finally, one of the strengths of our study is that the previously validated instrument [43] we used to examine Mediterranean diet adherence was created for the American firefighters, based on their lifestyle, eating habits, nature of work (meals at home and at work), type of drinks, and alcohol consumption and therefore is a good-quality validated instrument for this population, as it is known that the quality of Mediterranean diet scores has been questioned in different populations [38].

## 5. Conclusions

In conclusion, greater adherence to a Mediterranean diet, as measured by a higher mMDS, was favorably associated with lower measures of cardiometabolic risk. In fully adjusted models including physical activity level and smoking, the associations of a higher mMDS with a lower total cholesterol/HDL ratio and increased HDL-cholesterol remained robust. The mMDS has now evidence of validity with respect to more established questionnaires and has been determined in relation to additional biologically plausible associations from two different and independent mid-western (US) firefighter cohorts. Therefore, the mMDS should be a valid tool for assessing the outcome of cluster-randomized controlled trials of Mediterranean lifestyle interventions in this population and similar ones. It may also have further utility not only in research but also in clinical practice.

## Figures and Tables

**Table 1:** Characteristics of the participants.

Characteristic	*N*	
Male gender, *n* (%)	448	423 (94.4)
Age (years), mean (SD)	460	46.7 (8.3)
Race, *n* (%)	311	
Caucasian		266 (85.5)
African American		39 (12.5)
Other		6 (1.9)
Currently smoking, *n* (%)	314	15 (4.8)
Physical activity *, *n* (%)	307	
Low		39 (12.7)
Medium		65 (21.2)
High		203 (66.1)
Hours sitting per week, median (Q1–Q3)	300	15 (10–24)
Number of meals at the firehouse, median (Q1–Q3)	309	3 (2–3)
FFQ mMDS, mean (SD)	460	21.88 (6.68)
Anthropometric variables		
BMI (kg/m^2^), mean (SD)	460	30.01 (4.39)
Normal weight	74	16%
Overweight	156	34%
Obese	230	50%
Waist circumference (cm), mean (SD)	459	99.7 (12.5)
Body fat percentage (%), mean (SD)	458	28.10 (6.55)
Blood pressure variables		
Resting SBP (mmHg), mean (SD)	460	125.5 (11.2)
Resting DBP (mmHg), mean (SD)	460	79.1 (6.8)
Biochemical variables		
Total Cholesterol (mg/dL), mean (SD)	460	197.1 (37.7)
HDL-Cholesterol (mg/dL), mean (SD)	460	48.5 (11.4)
LDL-Cholesterol (mg/dL), mean (SD)	452	123.5 (32.6)
Total Cholesterol/HDL ratio, mean (SD)	460	4.26 (1.32)
Triglycerides (mg/dL), mean (SD)	459	126.0 (76.6)
Glucose (mg/dL), mean (SD)	460	99.5 (19.6)

* Physical activity. Low: did not participate regularly in programmed recreation, sport, or heavy physical activity. Medium: participated regularly in recreation requiring modest physical activity, such as golf, horseback riding, calisthenics, gymnastics, table tennis, bowling, weight-lifting, yard work. High: participated regularly in heavy physical exercise such as running or jogging, swimming, rowing, skipping rope, running in place, or engaging in vigorous aerobic activity such as tennis. basketball, or handball. FFQ: Food Frequency questionnaire, mMDS: modified Mediterranean Diet Score, SD: Standard Deviation, BMI: body mass index, SBP: systolic blood pressure, DBP: diastolic blood pressure, HDL: high-density lipoprotein, LDL: low-density lipoprotein.

**Table 2:** Association of the mMDS (categorized into quartiles) with anthropometric measures, blood pressure, and biochemical variables.

	mMDS						
Risk Factor	1st Quartile	2nd Quartile	3rd Quartile	4th Quartile	*P* Trend *	*P* Trend ^†^	*P* Trend ^‡^
Number of subjects	106	122	118	114			
Anthropometric variables							
BMI (kg/m^2^)	30.59 (4.06)	30.14 (4.82)	30.17 (4.70)	29.16 (3.77)	0.023	0.030	0.914
Waist circumference (cm)	102.0 (11.6)	100.6 (13.6)	99.3 (12.9)	96.8 (11.0)	0.001	0.002	0.685
Body fat percentage (%)	28.96 (5.64)	28.61 (6.38)	28.42 (7.06)	26.42 (6.75)	0.005	0.002	0.886
Blood pressure variables							
Resting SBP (mmHg)	125.7 (10.8)	124.7 (11.4)	126.6 (12.7)	125.1 (9.5)	0.980	0.836	0.515
Resting DBP (mmHg)	79.6 (7.2)	78.8 (6.7)	79.3 (6.3)	78.6 (7.1)	0.418	0.522	0.927
Biochemical variables							
Total Cholesterol (mg/dL)	200.2 (36.6)	193.1 (37.0)	198.2 (41.4)	197.5 (35.4)	0.894	0.876	0.742
HDL-Cholesterol (mg/dL)	45.6 (10.1)	48.6 (11.9)	48.7 (11.5)	50.9 (11.4)	0.001	0.002	0.022
LDL-Cholesterol (mg/dL)	127.3 (32.7)	119.5 (30.1)	124.4 (35.3)	123.6 (32.2)	0.703	0.690	0.587
Total Cholesterol/HDL ratio	4.60 (1.31)	4.19 (1.58)	4.27 (1.28)	4.03 (0.96)	0.004	0.007	0.020
Triglycerides (mg/dL)	140.8 (85.9)	118.4 (62.7)	129.2 (88.4)	116.9 (65.7)	0.071	0.107	0.364
Glucose (mg/dL)	99.9 (14.3)	100.9 (23.2)	98.6 (20.5)	98.6 (18.9)	0.450	0.594	0.770

* unadjusted; ^†^ adjusted for gender and age; ^‡^ adjusted for age, gender, race, physical activity, and smoking.

**Table 3:** Effect of a unitary increase in the mMDS on anthropometric measures, blood pressure, and biochemical variables.

	Linear Regression Models					
	Adjusted by Gender and Age			Adjusted by Age, Gender, Race, Physical Activity, and Smoking		
Risk Factor	Β Coefficient	SE	*p* Value	Β Coefficient	SE	*p* Value
Anthropometric variables						
BMI (kg/m^2^)	−0.080	0.030	0.008	−0.026	0.038	0.490
Waist circumference (in)	−0.114	0.031	<0.001	−0.045	0.039	0.241
Body fat percentage (%)	−0.141	0.043	0.001	−0.028	0.057	0.627
Blood pressure variables						
Resting SBP (mmHg)	−0.041	0.076	0.590	0.004	0.107	0.969
Resting DBP (mmHg)	−0.056	0.046	0.223	−0.037	0.062	0.552
Biochemical variables						
Total Cholesterol (mg/dL)	−0.160	0.264	0.546	−0.289	0.332	0.385
HDL Cholesterol (mg/dL)	0.254	0.075	<0.001	0.286	0.100	0.004
LDL Cholesterol (mg/dL)	−0.193	0.230	0.402	−0.341	0.300	0.256
Total cholesterol-HDL ratio	−0.028	0.009	0.002	−0.030	0.010	0.002
Triglycerides (mg/dL)	−1.010	0.532	0.058	−0.909	0.644	0.159
Glucose (mg/dL)	−0.137	0.135	0.313	−0.155	0.186	0.404

SD, standard deviation; B, unstandardized Beta coefficient; SE, standard error.

## Appendix A

**Table A1:** Comparison of the single items of the modified Mediterranean diet scores (mMDS) according to biochemical indices.

	Total Cholesterol-HDL Ratio > 6					Triglycerides ≥ 150 mg/dL					Glucose ≥ 100 mg/dL				
	No (*N* = 374)		Yes (*N* = 27)		*p* Value	No (*N* = 300)		Yes (*N* = 100)		*p* Value	No (*N* = 243)		Yes (*N* = 158)		*p* Value
	Mean	SD	Mean	SD		Mean	SD	Mean	SD		Mean	SD	Mean	SD	
Total mMDS	22.23	6.80	18.19	6.63	0.003	22.47	6.80	20.44	6.86	0.010	22.12	6.67	21.70	7.15	0.549
Single item mMDS															
Fast food consumption *	1.57	0.95	1.00	0.83	0.003	1.66	0.93	1.14	0.92	<.001	1.51	0.97	1.56	0.93	0.634
Fruit consumption	1.57	0.90	1.22	0.70	0.049	1.58	0.91	1.44	0.82	0.163	1.58	0.94	1.49	0.80	0.270
Vegetable consumption	2.56	1.06	2.44	0.93	0.586	2.53	1.05	2.63	1.08	0.396	2.56	1.07	2.54	1.03	0.841
Sweet desserts consumption	1.85	1.57	0.96	1.22	0.004	1.93	1.57	1.37	1.50	0.002	1.76	1.57	1.84	1.56	0.625
Cooking oil or fat use at home	2.12	1.85	1.63	1.74	0.185	2.12	1.86	1.99	1.84	0.554	2.22	1.83	1.88	1.86	0.073
Fried food consumption	1.56	1.18	0.89	0.93	0.004	1.58	1.19	1.30	1.10	0.037	1.47	1.18	1.58	1.17	0.374
Breads or starches consumed at home	1.75	1.48	1.67	1.52	0.782	1.82	1.47	1.50	1.51	0.062	1.73	1.49	1.77	1.48	0.805
Ocean fish consumption	1.64	1.14	1.70	0.99	0.765	1.67	1.13	1.57	1.12	0.459	1.66	1.07	1.61	1.21	0.700
Non-alcoholic beverages at home	2.66	1.13	2.44	1.37	0.339	2.69	1.14	2.50	1.17	0.144	2.74	1.12	2.50	1.18	0.036
Alcoholic beverages	2.09	1.57	1.78	1.48	0.317	2.02	1.60	2.24	1.44	0.224	2.03	1.59	2.13	1.54	0.516
Wine consumption	0.81	0.98	0.89	1.01	0.679	0.81	0.98	0.82	0.99	0.953	0.83	0.99	0.78	0.98	0.645
Legumes consumption	0.67	1.26	0.74	1.29	0.791	0.66	1.26	0.74	1.29	0.585	0.71	1.29	0.63	1.23	0.510
Nuts consumption	1.39	1.62	0.81	1.44	0.073	1.40	1.62	1.20	1.61	0.277	1.32	1.59	1.41	1.66	0.612

* Fast-food consumption per week (score 0–4), i.e., frequency of choosing options such as McDonalds, Burger King, Kentucky Fried Chicken, etc.

## References

[1] Soares E.M.K.V.K., Smith D., Porto L.G.G. (2020). Worldwide prevalence of obesity among firefighters: A systematic review protocol. BMJ Open.

[2] Tsismenakis A.J., Christophi C.A., Burress J.W., Kinney A.M., Kim M., Kales S.N. (2009). The obesity epidemic and future emergency responders. Obesity.

[3] Lavie C.J., Milani R.V., Ventura H.O. (2009). Obesity and cardiovascular disease. risk factor, paradox, and impact of weight loss. J. Am. Coll. Cardiol..

[4] Dunlay S.M., Givertz M.M., Aguilar D., Allen L.A., Chan M., Desai A.S., Deswal A., Dickson V.V., Kosiborod M.N., Lekavich C.L. (2019). Type 2 diabetes mellitus and heart failure, a scientific statement from the American Heart Association and Heart Failure Society of America. J. Card. Fail..

[5] Donovan R., Nelson T., Peel J., Lipsey T., Voyles W., Israel R.G. (2009). Cardiorespiratory fitness and the metabolic syndrome in firefighters. Occup. Med..

[6] Soteriades E.S., Hauser R., Kawachi I., Liarokapis D., Christiani D.C., Kales S.N. (2005). Obesity and cardiovascular disease risk factors in firefighters: A prospective cohort study. Obes. Res..

[7] Navarro K.M., Kleinman M.T., Mackay C.E., Reinhardt T.E., Balmes J.R., Broyles G.A., Ottmar R.D., Naher L.P., Domitrovich J.W. (2019). Wildland firefighter smoke exposure and risk of lung cancer and cardiovascular disease mortality. Environ. Res..

[8] Kales S., Smith D.L. (2017). Firefighting and the heart. Circulation.

[9] Smith D.L., Haller J.M., Korre M., Fehling P.C., Sampani K., Porto L.G.G., Christophi C.A., Kales S.N. (2018). Pathoanatomic findings associated with duty-related cardiac death in US firefighters: A case-control study. J. Am. Heart Assoc..

[10] Smith D.L., Haller J.M., Korre M., Sampani K., Porto L.G.G., Fehling P.C., Christophi C.A., Kales S.N. (2019). The relation of emergency duties to cardiac death among US firefighters. Am. J. Cardiol..

[11] Kahn S.A., Leonard C., Siordia C. (2018). Firefighter fatalities: Crude mortality rates and risk factors for line of duty injury and death. J. Burn Care Res..

[12] Gao X., Deming N.J., Moore K., Alam T. (2020). Cardiorespiratory fitness decline in aging firefighters. Am. J. Public Health.

[13] Neovius M., Kark M., Rasmussen F. (2008). Association between obesity status in young adulthood and disability pension. Int. J. Obes..

[14] Linde J.A., Andrade K., MacLehose R.F., Mitchell N.R., Harnack L., Cousins J.M., Graham D.J., Jeffery R.W. (2012). HealthWorks: Results of a multi-component group-randomized worksite environmental intervention trial for weight gain prevention. Int. J. Behav. Nutr. Phys. Act..

[15] Baur D.M., Christophi C.A., Cook E.F., Kales S. (2012). Age-Related decline in cardiorespiratory fitness among career firefighters: Modification by physical activity and adiposity. J. Obes..

[16] Eleftheriou D., Benetou V., Trichopoulou A., La Vecchia C., Bamia C. (2018). Mediterranean diet and its components in relation to all-cause mortality: Meta-analysis. Br. J. Nutr..

[17] Barbagallo M., Barbagallo M. (2004). Mediterranean diet and longevity. Eur. J. Cancer Prev..

[18] Bo S., Ponzo V., Goitre I., Fadda M., Pezzana A., Beccuti G., Gambino R., Cassader M., Soldati L., Broglio F. (2016). Predictive role of the Mediterranean diet on mortality in individuals at low cardiovascular risk: A 12-year follow-up population-based cohort study. J. Transl. Med..

[19] Korre M., Tsoukas M.A., Frantzeskou E., Yang J., Kales S. (2014). Mediterranean diet and workplace health promotion. Curr. Cardiovasc. Risk Rep..

[20] Nissensohn M., Román-Viñas B., Sánchez-Villegas A., Piscopo S., Serra-Majem L. (2016). The Effect of the Mediterranean diet on hypertension: A systematic review and meta-analysis. J. Nutr. Educ. Behav..

[21] Korre M., Sotos-Prieto M., Kales S. (2017). Survival Mediterranean style: Lifestyle changes to improve the health of the US fire service. Front. Public Health.

[22] Benhammou S., Heras-González L., Ibáñez-Peinado D., Barceló C., Hamdan M., Rivas A., Mariscal-Arcas M., Olea-Serrano F., Monteagudo C. (2016). Comparison of Mediterranean diet compliance between European and non-European populations in the Mediterranean basin. Appetite.

[23] Dinu M., Pagliai G., Casini A., Sofi F. (2018). Mediterranean diet and multiple health outcomes: An umbrella review of meta-analyses of observational studies and randomised trials. Eur. J. Clin. Nutr..

[24] Malakou E., Linardakis M., Armstrong M.E., Zannidi D., Foster C., Johnson L., Papadaki A. (2018). The combined effect of promoting the Mediterranean diet and physical activity on metabolic risk factors in adults: A systematic review and meta-analysis of randomised controlled trials. Nutrients.

[25] Sotos-Prieto M., Moreno-Franco B., Ordovás J.M., León M., Casasnovas J.A., Peñalvo J.L. (2015). Design and development of an instrument to measure overall lifestyle habits for epidemiological research: The Mediterranean Lifestyle (MEDLIFE) index. Public Health Nutr..

[26] Della Corte C., Mosca A., Vania A., Alterio A., Iasevoli S., Nobili V. (2017). Good adherence to the Mediterranean diet reduces the risk for NASH and diabetes in pediatric patients with obesity: The results of an Italian Study. Nutrition.

[27] Serra-Majem L., Román-Viñas B., Sanchez-Villegas A., Guasch-Ferré M., Corella D., La Vecchia C. (2019). Benefits of the Mediterranean diet: Epidemiological and molecular aspects. Mol. Asp. Med..

[28] Foscolou A., Magriplis E., Tyrovolas S., Soulis G., Bountziouka V., Mariolis A., Piscopo S., Valacchi G., Anastasiou F., Gotsis E. (2018). Lifestyle determinants of healthy ageing in a Mediterranean population: The multinational MEDIS study. Exp. Gerontol..

[29] Izadi V., Tehrani H., Haghighatdoost F., Dehghan A., Surkan P.J., Azadbakht L. (2016). Adherence to the DASH and Mediterranean diets is associated with decreased risk for gestational diabetes mellitus. Nutrition.

[30] Panagiotakos D.B., Georgousopoulou E.N., Pitsavos C., Chrysohoou C., Metaxa V., Georgiopoulos G., Kalogeropoulou K., Tousoulis D., Stefanadis C. (2015). Ten-Year (2002–2012) cardiovascular disease incidence and all-cause mortality, in urban Greek population: The ATTICA Study. Int. J. Cardiol..

[31] Panagiotakos D.B., Pitsavos C., Stefanadis C. (2006). Dietary patterns: A Mediterranean diet score and its relation to clinical and biological markers of cardiovascular disease risk. Nutr. Metab. Cardiovasc. Dis..

[32] Trichopoulos D. (2003). Adherence to a Mediterranean diet and survival in a Greek population. N. Engl. J. Med..

[33] Naska A., Trichopoulou A. (2014). Back to the future: The Mediterranean diet paradigm. Nutr. Metab. Cardiovasc. Dis..

[34] Gnagnarella P., Dragà D., Misotti A.M., Sieri S., Spaggiari L., Cassano E., Baldini F., Soldati L., Maisonneuve P. (2018). Validation of a short questionnaire to record adherence to the Mediterranean diet: An Italian experience. Nutr. Metab. Cardiovasc. Dis..

[35] Abu-Saad K., Endevelt R., Goldsmith R., Shimony T., Nitsan L., Shahar D.R., Keinan-Boker L., Ziv A., Kalter-Leibovici O. (2019). Adaptation and predictive utility of a Mediterranean diet screener score. Clin. Nutr..

[36] Guasch-Ferré M., Salas-Salvadó J., Ros E., Estruch R., Corella D., Fitó M., Martinez-Gonzalez M., Arós Borau F., Gómez-Gracia E., Fiol M. (2017). The PREDIMED trial, Mediterranean diet and health outcomes: How strong is the evidence?. Nutr. Metab. Cardiovasc. Dis..

[37] Martínez-González M.Á., García-Arellano A., Toledo E., Salas-Salvadó J., Buil-Cosiales P., Corella D., Covas M.I., Schröder H., Arós F., Gómez-Gracia E. (2012). A 14-Item Mediterranean diet assessment tool and obesity indexes among high-risk subjects: The PREDIMED Trial. PLoS ONE.

[38] Zaragoza-Martí A., Cabañero-Martínez M.J., Hurtado-Sánchez J.A., Laguna-Pérez A., Ferrer-Cascales R. (2018). Evaluation of Mediterranean diet adherence scores: A systematic review. BMJ Open.

[39] Yang J., Farioli A., Korre M., Kales S.N. (2014). Modified Mediterranean Diet score and cardiovascular risk in a north American working population. PLoS ONE.

[40] Sotos-Prieto M., Cash S.B., Christophi C., Folta S.C., Moffatt S., Muegge C.M., Korre M., Mozaffarian D., Kales S.N. (2017). Rationale and design of feeding America’s bravest: Mediterranean diet-based intervention to change firefighters’ eating habits and improve cardiovascular risk profiles. Contemp. Clin. Trials.

[41] Willett W.C., Sampson L., Stampfer M.J., Rosner B., Bain C., Witschi J., Hennekens C.H., Speizer F.E. (1985). Reproducibility and validity of a semiquantitative food frequency questionnaire. Am. J. Epidemiol..

[42] Salvini S., Hunter D.J., Sampson L., Stampfer M.J., Colditz G.A., Rosner B., Willett W.C. (1989). Food-Based validation of a dietary questionnaire: The effects of week-to-week variation in food consumption. Int. J. Epidemiol..

[43] Sotos-Prieto M., Christophi C., Black A., Furtado J.D., Song Y., Magiatis P., Papakonstantinou A., Melliou E., Moffatt S., Kales S.N. (2019). Assessing validity of self-reported dietary intake within a Mediterranean diet cluster randomized controlled trial among US firefighters. Nutrients.

[44] Jackson A.S., Blair S.N., Mahar M.T., Wier L.T., Ross R.M., Stuteville J.E. (1990). Prediction of functional aerobic capacity without exercise testing. Med. Sci. Sports Exerc..

[45] Hershey M.S., Sotos-Prieto M., Ruiz-Canela M., Martínez-González M.Á., Cassidy A., Moffatt S., Kales S. (2020). Anthocyanin intake and physical activity: Associations with the lipid profile of a US working population. Molecules.

[46] Estruch R. (2010). Anti-Inflammatory effects of the Mediterranean diet: The experience of the PREDIMED study. Proc. Nutr. Soc..

[47] Amato M., Bonomi A., Laguzzi F., Veglia F., Tremoli E., Werba J.P., Giroli M.G. (2020). Overall dietary variety and adherence to the Mediterranean diet show additive protective effects against coronary heart disease. Nutr. Metab. Cardiovasc. Dis..

[48] Merino J., Kones R., Ros E. (2018). Effects of Mediterranean diet on endothelial function. Endothelium and Cardiovascular Diseases.

[49] Romaguera D., Norat T., Mouw T., May A.M., Bamia C., Slimani N., Travier N., Besson H., Luan J., Wareham N. (2009). Adherence to the Mediterranean Diet is associated with lower abdominal adiposity in European men and women. J. Nutr..

[50] Mattei J., Sotos-Prieto M., Bigornia S.J., Noel S.E., Tucker K.L. (2017). The Mediterranean diet score is more strongly associated with favorable cardiometabolic risk factors over 2 years than other diet quality indexes in puerto rican adults. J. Nutr..

[51] Aoun C., Papazian T., Helou K., El Osta N., Khabbaz L.R. (2019). Comparison of five international indices of adherence to the Mediterranean diet among healthy adults: Similarities and differences. Nutr. Res. Pract..

[52] Trichopoulou A., Naska A., Orfanos P., Trichopoulos D. (2005). Mediterranean diet in relation to body mass index and waist-to-hip ratio: The Greek European prospective investigation into cancer and nutrition study. Am. J. Clin. Nutr..

[53] Estruch R., Martínez-González M.A., Corella D., Salas-Salvadó J., Ruiz-Gutiérrez V., Covas M.I., Fiol M., Gómez-Gracia E., López-Sabater M.C., Vinyoles E. (2006). Effects of a Mediterranean-style diet on cardiovascular risk factors a randomized trial. Ann. Intern. Med..

[54] Estruch R., Ros E., Salas-Salvadó J., Covas M.-I., Corella D., Arós F., Gómez-Gracia E., Ruiz-Gutiérrez V., Fiol M., Lapetra J. (2013). Primary prevention of cardiovascular disease with a Mediterranean diet. N. Engl. J. Med..

[55] Sahingoz S.A., Sanlier N. (2011). Compliance with Mediterranean Diet Quality Index (KIDMED) and nutrition knowledge levels in adolescents. A case study from Turkey. Appetite.

[56] Krauss R.M., Eckel R.H., Howard B., Appel L.J., Daniels S.R., Deckelbaum R.J., Erdman J.W., Kris-Etherton P., Goldberg I.J., Kotchen T.A. (2000). AHA Dietary Guidelines: Revision 2000: A statement for healthcare professionals from the Nutrition Committee of the American Heart Association. Circulation.

[57] Willett W.C. (2006). The Mediterranean diet: Science and practice. Public Health Nutr..

[58] Mozaffarian D. (2016). Dietary and policy priorities for cardiovascular disease, diabetes, and obesity. Circulation.

[59] Martínez-González M.A., Hershey M.S., Zazpe I., Trichopoulou A. (2017). Transferability of the Mediterranean diet to non-mediterranean countries. What is and what is not the Mediterranean diet. Nutrients.

[60] Soriguer F., Rojo-Martínez G., Dobarganes M.C., Almeida J.M.G., Esteva I., Beltrán M., De Adana M.S.R., Tinahones F., Gómez-Zumaquero J.M., García-Fuentes E. (2003). Hypertension is related to the degradation of dietary frying oils. Am. J. Clin. Nutr..

[61] Godos J., Rapisarda G., Marventano S., Galvano F., Mistretta A., Grosso G. (2017). Association between polyphenol intake and adherence to the Mediterranean diet in Sicily, southern Italy. NFS J..

[62] Pérez-Martínez P., Mikhailidis D.P., Athyros V.G., Bullo M., Couture P., Covas M.I., De Koning L., Delgado-Lista J., Díaz-López A., Drevon C.A. (2017). Lifestyle recommendations for the prevention and management of metabolic syndrome: An international panel recommendation. Nutr. Rev..

[63] Morin S.J., Gaziano J.M., Djoussé L. (2017). Relation between plasma phospholipid oleic acid and risk of heart failure. Eur. J. Nutr..

[64] Gurevich K., Poston W.S.C., Anders B., Ivkina M.A., Archangelskaya A., Jitnarin N., Starodubov V.I. (2016). Obesity prevalence and accuracy of BMI-defined obesity in Russian firefighters. Occup. Med..

